# KOH activation of carbon electrodes for enhanced capacitive dechlorination: Performance and mechanisms

**DOI:** 10.1371/journal.pone.0347780

**Published:** 2026-05-27

**Authors:** Jianna Jia, Chang Lu, Jinlong Han, Wenrui Wang, Kailei Zhang, Zhijun Ren

**Affiliations:** 1 Tianjin Research Institute for Water Transport Engineering, M.O.T., Tianjin, China; 2 School of Energy and Environmental Engineering, Hebei University of Technology, Tianjin, China; National Chung Cheng University, Taiwan & Australian Center for Sustainable Development Research and Innovation (ACSDRI), AUSTRALIA

## Abstract

Coal transportation terminals produce wastewater containing high concentrations of chloride, which can cause corrosion of equipment and be hazardous to the environment. Capacitive deionisation (CDI) has been discussed as an alternative to conventional dechlorination methods, given that it consumes low energy and avoids secondary pollution. This paper involved the development of a KOH-modified activated carbon (KOH-AC) electrode, which must be used to improve the chloride removal performance of CDI. The obtained characterization results showed that, under the activation of KOH, the specific surface area of activated carbon (104.48 m^2^/g to 1390.88 m^2^/g) was greatly intensified, the pore structure became more uniform, and plentiful oxygen-containing functional groups were formed, improving electrochemical activity and ion adsorption capacity. In optimal operating conditions (1.2 V, 20 mL/min), the KOH-AC electrode obtained a chloride ion removal efficiency of more than 80% and was extremely stable even after numerous adsorption-desorption cycles. Moreover, the CatBoost exhibited high accuracy in predicting chloride removal efficiency (R^2^ = 0.9114). The shap analysis revealed that the flow rate and influent NaCl concentration were the most significant factors for dechlorination. This study provides insights for the efficient removal of chlorinated wastewater in coal transportation terminals, while the constructed model offers a reference for precise treatment.

## 1. Introduction

As a pivotal node for coal transportation, coal terminals are subjected to elevated levels of salt minerals and seawater intrusion over an extended period, leading to a substantial accumulation of chloride ions [1,2]. Residual chloride ions have been shown to be the primary cause of chemical corrosion in metal pipelines, loading and unloading equipment, and wastewater treatment system. The corrosion rate is reported to range from 0.5 to 1.2 mm/year, which has been shown to shorten the lifespan of the equipment by 30% to 50% and to increase the risk of leakage, thereby triggering secondary pollution [3]. It is important to note that chlorine wastewater infiltration into the soil will exacerbate salinization, damage soil structure, and inhibit crop nutrient absorption [4]. Furthermore, chlorine reacts with organic matter to form chlorinated by-products, which pose a serious threat to aquatic organisms and human health [5]. Consequently, effective dechlorination is imperative for ensuring harbour safety, ecological health and sustainable development.

Conventional dechlorination technologies encompass ion exchange, reverse osmosis and chemical precipitation [6]. However, these processes are inherently limited in their efficacy. Specifically, the ion exchange process necessitates more frequent regeneration of the resin, resulting in increased operating costs [7], while reverse osmosis technology is confronted with challenges such as membrane contamination and high energy consumption [8]. Furthermore, chemical precipitation methods are vulnerable to fluctuations in water quality during the process of dechlorination [9]. Conversely, capacitive deionization (CDI) has been identified as a highly effective and rapid electrochemical desalination process, rendering it well-suited for the treatment of highly chlorinated wastewater. This is due to the fact that it exhibits no secondary pollution, has minimal energy consumption and straightforward operational characteristics [10,11]. Datar et al. [12] explained that CDI uses the double-layer capacitance principle to achieve dechlorination by moving chloride ions to the anode through a low voltage direct current (DC) of 1–2 V. Hui et al. [13] reported that the CDI system is well suited for high concentration chlorinated wastewater. Therefore, CDI is expected to address the challenges of large fluctuations, both in terms of chloride ion concentrations and in terms of the complexity of the water quality, in coal transportation terminal wastewater.

Being the core parts of CDI systems, electrode materials control the dechlorination performance, adsorption capacity, and cycling stability [13]. Activated carbon (AC) serves as an electrode material in CDI systems, offering advantages such as a porous structure, high conductivity, and environmental friendliness [14]. Nevertheless, pristine AC has a non-uniform distribution of pores, low surface characteristics, and low regeneration stability [15]. To address this issue, structural and surface modifications have become research hotspot. Principal strategies include [16–18]: (i) physical methods, (ii) chemical methods, and (iii) composite methods. In complex environments such as coal terminals, alkaline modification emerges as a highly attractive wastewater treatment method [19]. Han et al. [20] indicated that the activation of KOH effectively increased the specific surface area of activated carbon. This is primarily due to the expansion of the pore structure, which simultaneously introduces a large number of oxygen-containing functional groups, significantly enhancing the adsorption capacity for ions [21,22].

To better understand the effect of activated carbon on CDI dechlorination, machine learning provides an effective and innovative approach [23]. Machine learning models are capable of handling massive amounts of data and capturing complex and nonlinear relationships between factors [24,25]. This capability enables accurate prediction of dechlorination performance and opens up new pathways for efficient dechlorination optimization in coal terminals. By applying models such as Random Forest, Extreme Gradient Enhancement, and Gradient Boosting, researchers can predict the effects of different factor conditions on CDI dechlorination, including activated carbon materials, influent water conditions, and electrode modulation [26,27]. In addition, machine learning algorithms can help identify and rank the most influential factors, guiding researchers to improve the performance of CDI dichlorination [28].

Therefore, to address the demand for efficient dechlorination of wastewater from coal transport terminals, and based on a CDI process predominantly governed by electric double-layer (EDL) formation under low-voltage polarization rather than new Faradaic reaction pathways, the following research was conducted: (1) Preparation of novel modified activated carbon electrodes; (2) Elucidation of the influence of modified electrodes on CDI electrosorption performance under different conditions; (3) Establishment of machine learning models to predict CDI electrosorption performance.

## 2. Materials and methods

### 2.1 Experimental reagents

All reagents used were commercially available, see details in S1 Text.

### 2.2 Capacitive deionization device

**Fig 1:**
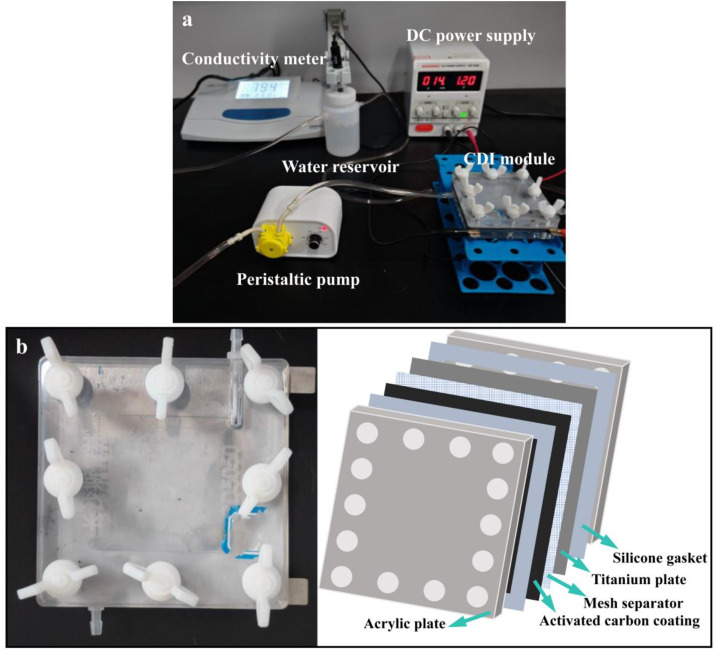
(a) Electrosorption experimental device; (b) CDI module.

Capacitive deionization (CDI) experiments were conducted using a custom-built system consisting of a DC power supply, peristaltic pump, conductivity meter, feed reservoir, and a CDI module (Type CD5050, effective volume 25 cm^3^; Fig 1a). The CDI cell (9 × 9 × 3.5 cm) had an internal volume of 7.5 mL and was operated under constant voltage supplied by the DC power source to drive the electrosorption desalination process. As the core component (Fig 1b), the module enabled reversible ion adsorption and desorption. It comprised acrylic plates, silicone gaskets, activated carbon (AC) coatings, mesh separators, and titanium plate current collectors (S1 Table). The distance between the positive and negative electrodes was fixed at 2 mm to ensure stable electric field distribution. The acrylic plates provided structural support, silicone gaskets ensured sealing and controlled electrode spacing, titanium plates guaranteed uniform current distribution, and mesh separators prevented electrical short circuits. During operation, 150 mL of electrolyte solution was circulated through the module via M5–3.8 liquid pipe joints, and conductivity was continuously monitored in real time. Adsorption equilibrium was defined when the conductivity stabilized. Ion desorption was subsequently achieved by polarity reversal or short-circuiting. Cycling tests were conducted to evaluate electrode stability, and all experiments were performed at room temperature.

The primary pollutant in the simulated coal terminal wastewater was chloride. The synthetic wastewater was prepared by dissolving sodium chloride (NaCl) in deionized water. To investigate the effect of salinity, NaCl concentrations ranging from 100 to 1000 mg/L were used. The solution pH was adjusted to 7.0 ± 0.2 using negligible volumes of 0.1 M HCl or NaOH. The conductivity of each solution was measured at 25°C using a calibrated conductivity meter. A strong linear correlation (R^2^ > 0.99) was observed between NaCl concentration and conductivity within the tested range.

### 2.3 KOH-AC electrode preparation

The AC powder used was lab-synthesized by carbonizing corn straw at 600°C under N_2_ atmosphere for 90 minutes. The obtained AC powder was first sieved through a 0.05 mm (300-mesh) screen to remove large particles, then repeatedly washed with deionized water until the effluent conductivity fell below 10 μS/cm, and finally dried at 120 °C for later use. The dried AC was mixed with KOH at a mass ratio of 1:2, and a small amount of deionized water was added to dissolve the mixture. After vigorous stirring for 2 h and ultrasonication for 0.5 h, the mixture was dried at 60 °C for 12 h. Once completely dried, it was heated under a nitrogen atmosphere to 800 °C at a ramp rate of 5 °C/min and held for 2 h. The resulting activated product was soaked and rinsed repeatedly with deionized water until neutrality, followed by drying to obtain KOH-modified activated carbon (KOH-AC).

The activated carbon was then mixed with conductive carbon black at a mass ratio of 75:15. A polytetrafluoroethylene (PTFE) emulsion was added as a binder, and N-methyl-2-pyrrolidone was used as the solvent. The mixture was magnetically stirred for 12 h to form a homogeneous slurry. The coated electrode was dried at 80 °C for 12 hours in a vacuum oven to remove the solvent. Finally, the dried electrode was pressed under 10 MPa for 1 minute to enhance the contact between the active material and the current collector. The final thickness of the electrode film after pressing is approximately 1 mm. The electrodes were prepared using exactly the same procedure to ensure the symmetry of the CDI cell.

The active material mass loading on a single electrode was controlled to be approximately 1.53 g. The resulting electrodes were immersed in 1.5 mol/L KOH for 24 h (with precautions to avoid air exposure), thoroughly rinsed with deionized water, and dried at 70 °C for 6 h in a forced-air oven. A 60% PTFE emulsion was used as the binder. To optimize the binder formulation, different solid contents of 60% PTFE emulsion (4%, 6%, 8%, 10%, 12%) were tested (S1 Fig). When the PTFE content reached 8%, the electrode exhibited the lowest conductivity, indicating reduced electron transfer, while achieving the highest salt removal performance, so this ratio was chosen.

### 2.4 Conversion between conductivity and Cl^-^ concentration

To evaluate the dechlorination performance of the electrosorption system, this study employed an electrosorption desalination device, with the desalination efficiency assessed indirectly by monitoring changes in the conductivity of the inlet and outlet solutions. Due to the complexity of direct salt concentration measurements, solution conductivity was selected as the performance evaluation indicator, as it exhibits a strong linear correlation with ion concentration within a certain range [5]. Sodium chloride solutions with concentrations ranging from 100 to 1000 mg/L were prepared as simulated saline water. The conductivity of these solutions was measured at 25°C, and the results were linearly fitted, as summarized in S2 Table and S2 Fig. The linear regression equation for Cl^-^ versus conductivity is as follows:


$$\begin{array}{c} y=1.6406x+48.326 \end{array}$$

where: y represents electrical conductivity (μs/cm), x represents Cl^-^ concentration (mg/L), and the correlation coefficient R^2^ = 0.9973.

### 2.5 Analytical methods

Scanning electron microscopy (SEM, ZEISS Sigma 360, Germany) was used to analyse the surface morphology of the activated carbon. The Brunauer-Emmett-Teller (BET) method was used to identify the specific surface area and pore size distribution (Micromeritics ASAP 2460, USA). X-ray diffraction (XRD Rigaku SmartLab SE, Japan) was used to determine the main components and the crystal structure, and Fourier transform infrared spectroscopy (FT-IR, Thermo Scientific Nicolet iS20, USA) was used to identify surface functional groups. The electrochemical activity and ion transport properties of the activated carbon were assessed by cyclic voltammetry (CV, scanning rate range: 5–125 mV/s) and electrochemical impedance spectroscopy (EIS) of the using an electrochemical work station (CHI660F, CH Instruments, USA).

### 2.6 Machine learning model analysis

According to the recently published literature related to the subject of electrosorption materials as used to remove chloride ion removal (retrieved from the Web of Science database), use Engage Digitizer software (https://engauge-digitizer.updatestar.com/) to extract literature data and create a chloride ion removal dataset. Four input parameters were chosen, including influent characteristics, and the removal efficiency of chloride ions was taken as the output parameter. The dataset was randomly split into training and testing sets at an 80:20 ratio. The former is used for model training and parameter optimization, while the latter is used to validate performance. Missing datas were imputed using the k-nearest neighbors’ algorithm (KNN, k = 5). Six machine learning models (RF, CatBoost, AdaBoost, XGBoost, HGBoost, and GBDT) were applied to systematically evaluate the predictive performance. All models were trained and tested in a Python 3.9 environment.

## 3. Results and discussion

### 3.1 Characterization and adsorption properties of KOH-AC

#### 3.1.1 Morphology.

**Fig 2:**
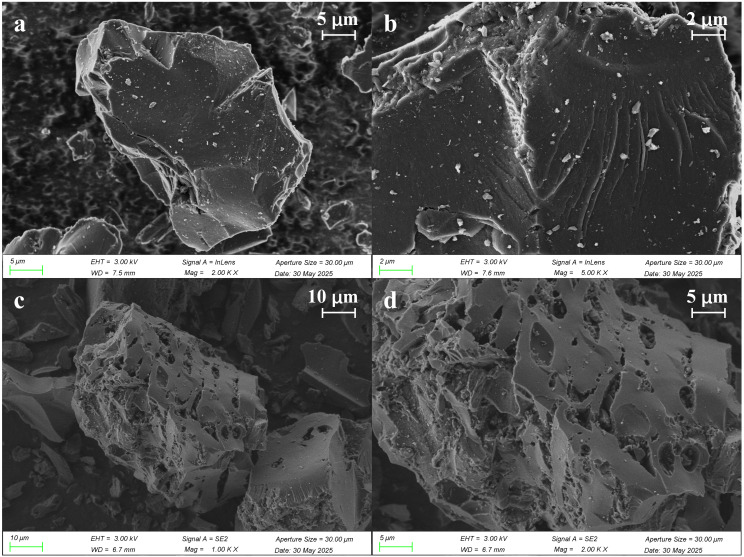
Activated carbon morphology before and after modification: (a-b) AC; (c-d) KOH-AC.

As observed from the surface morphology of the AC electrode (Fig 2), KOH modification significantly influences its surface structure. The AC surface was smooth and dense with no obvious pore structure (Fig 2a and 2b). After modification by KOH, the surface underwent significant fracturing, showing a rough porous morphology with a significant increase in the number and size of pores (Fig 2c and 2d). This transformation stems from the violent reaction of KOH with activated carbon during the activation process at 800°C. During this process, K_2_CO_3_ and K_2_O intermediate compounds were generated, while CO_2_, CO and H_2_ were released, which together contribute to the chemical corrosion of the carbon matrix [19]. When the temperature exceeded 765 °C, monatomic potassium evaporated and escaped, leading to pore deformation and pore collapse, and eventually forming a well-developed hierarchical pore structure [29]. In addition, the enhanced mesoporosity not only enlarges the electrode-electrolyte contact area, but also significantly improves the ion transport and adsorption capacity, effectively reducing the ion diffusion resistance and enabling the electrode to effectively capture salt ions for desalination purposes.

#### 3.1.2 Adsorption performance.

**Fig 3:**
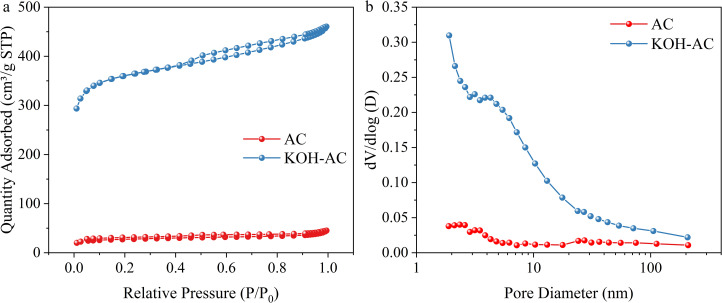
Pore size distribution curves derived from the BJH method: (a) nitrogen adsorption-desorption curve; (b) pore size distribution.

In order to compare the pore structures of AC and KOH-AC, nitrogen adsorption-desorption isotherms were used for the analysis. The adsorption capacity of AC was lower and the curve was relatively flat, whereas KOH-AC exhibited a higher adsorption capacity, and the curves showed the typical characteristics of a type IV isotherm (Fig 3a). The rapid increase in the KOH-AC curve at P/P_0_ < 0.1 indicated significant expansion of the micropores, which was consistent with the large microporous area (985.51 m^2^/g) obtained from t-plot analysis (Table 1). These abundant micropores are essential for CDI, as they provide the primary sites for electromigration and ion trapping within the electrical double layer (EDL) [30]. A significant hysteresis loop appeared in the P/P_0_ = 0.4–0.9 interval, reflecting the occurrence of capillary condensation in the range of slit-like pores and mesopores formed by stacked carbon layers [31]. It is noteworthy that when P/P_0_ > 0.9 still shows an increasing trend and no saturation occurs. This indicates the presence of macropores in addition to micropores and mesopores, which together constitute the graded pore channels [32]. This hierarchical architecture bridges macro-scale transport and micro-scale adsorption, where macropores and mesopores act as ‘ion highways’ to accelerate mass diffusion and electrosorption kinetics [28]. Overall, KOH-AC provided unobstructed channels for ion transport and mass diffusion.

**Table 1:** Specific surface area and pore structure parameters of AC and KOH-AC.

Sample	BET surface area (m^2^/g)	Micropore area (m^2^/g)	External surface area (m^2^/g)	Pore volume (cm^3^/g)	Adsorption surface area (m^2^/g)	Desorption surface area (m^2^/g)
AC	104.48	55.94	48.54	0.0219	25.43	26.26
KOH-AC	1390.88	985.51	405.37	0.3846	197.38	224.66

The pore size distribution (PSD) curve, calculated from the adsorption branch using the Barrett-Joyner-Halenda (BJH) model, is presented in Fig 3b. According to the pore size distribution, AC showed a multi-peak distribution, located at 2.3 nm, 3.2 nm and 8.5 nm, respectively. However, the PSD of KOH-AC exhibited a sharp, unimodal peak centered at approximately 4.3 nm, indicating a uniform mesoporous structure. In contrast, the PSD of pristine AC is broad and multimodal, indicating that KOH activation significantly enhanced the uniformity of the pore structure of the activated carbon. Such an optimized PSD is highly beneficial for CDI, as uniform mesopores reduce the steric hindrance and transport resistance of hydrated ions during high-speed migration [23]. It is noteworthy that the specific surface area, microporous area and external specific surface area of AC were 104.48 m^2^/g, 55.94 m^2^/g and 48.54 m^2^/g, respectively. The specific surface area, microporous area and external surface area were significantly increased by KOH-AC, reaching 1390.88 m^2^/g, 985.51 m^2^/g and 405.37 m^2^/g, respectively (Table 1). Meanwhile, the cumulative adsorption and desorption surface area of KOH-AC also increased dramatically, reaching 197.38 m^2^/g and 224.66 m^2^/g, respectively. Williams et al. [19] pointed out that during the high-temperature activation process of KOH, it reacted with the activated carbon to form gases such as CO_2_, CO, and K_2_CO_3_ to corrode and reconfigure the carbon skeleton, which in turn facilitated the development of the micropore and mesopore. The increased SSA and pore volume synergistically provide more accessible adsorption sites and prevent pore blockage [13]. Overall, KOH modification optimized the pore structure of activated carbon, resulting in a significant improvement in the SSA, pore volume and PSD, which synergistically contributes to a superior salt adsorption capacity and rapid desalination rate.

#### 3.1.3 Crystal structure and surface chemistry evolution after KOH activation.

**Fig 4:**
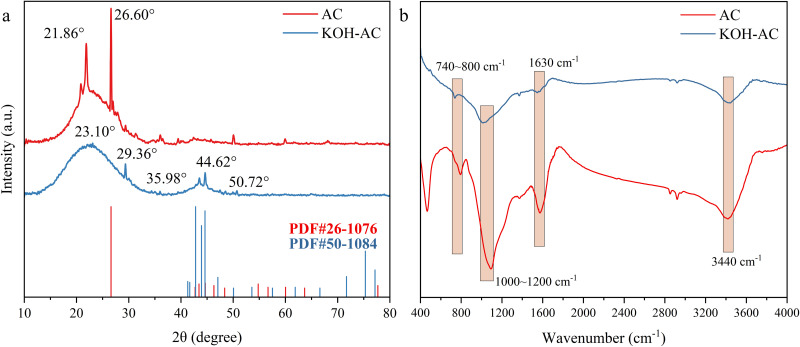
Characterization of different activated carbons: (a) XRD spectrum and (b) FTIR spectrum.

Various characterization methods were employed to study the structural evolution and surface chemical properties of AC and KOH-AC. The XRD pattern of AC exhibited characteristic diffraction peaks at 2θ = 21.86° and 26.60°, corresponding to interplanar spacings of 0.41 nm and 0.34 nm, respectively (Fig 4a). The AC is primarily composed of amorphous carbon and contains a small amount of graphite microcrystals [31]. In contrast, KOH-AC exhibited diffraction peaks at 2θ = 23.10°, 29.36°, 44.62°, and 50.72°, corresponding to interplanar spacings of 0.38 nm, 0.30 nm, 0.20 nm, and 0.18 nm, respectively. The main diffraction peak shifted from 21.86° to 23.10°, corresponding to a shortening of the interplanar spacing by 7.8%. These observations revealed that KOH-AC decreased the interlayer spacing and crystalline domain size while increasing structural disorder. This was attributed to the redox reactions of KOH and carbon, which cause the formation of metallic potassium and potassium carbonate [33]. Metallic potassium intercalates between carbon interlayers, causing structuring, expansion and reorganization at high temperatures. Subsequently, the carbon framework gradually collapses during evaporation and etching, creating large quantities of micropores and defective structures [19]. This results in interplanar spacing contraction and diffraction peak broadening. Additionally, the new diffraction peaks appearing in KOH-AC within the 29°-45° range may originate from localized microcrystalline graphitization or the formation of oxidized carbon phases [31].

FT-IR analysis further revealed significant differences in surface functional groups between AC and KOH-AC (Fig 4b). The intensified broad band at 3440 cm^-1^, assigned to O–H stretching vibrations, indicates increased hydroxyl groups and enhanced surface hydrophilicity, which facilitates electrolyte wetting and ion accessibility. The strengthened peaks at 1630 and 1400 cm^-1^ correspond to C = O and –COO^-^/C–OH functional groups, confirming the enrichment of oxygen-containing functionalities after activation [32]. Meanwhile, the enhanced aromatic C = C vibration at 1580 cm^-1^ and the attenuation of aliphatic C–H bands suggest promoted aromatization and partial graphitization of the carbon matrix [34,35], contributing to improved electronic conductivity. The combined effects of increased surface oxygen functionalities and structural ordering not only enhance charge transport but also influence chloride ion adsorption behavior [36]. Electrochemical polarization induces Cl^-^ accumulation primarily via electric double-layer formation in hierarchical pores, while surface oxygen-containing groups facilitate additional electrostatic and weak complexation interactions, leading to a coupled physical-chemical adsorption mechanism [37].

**Fig 5:**
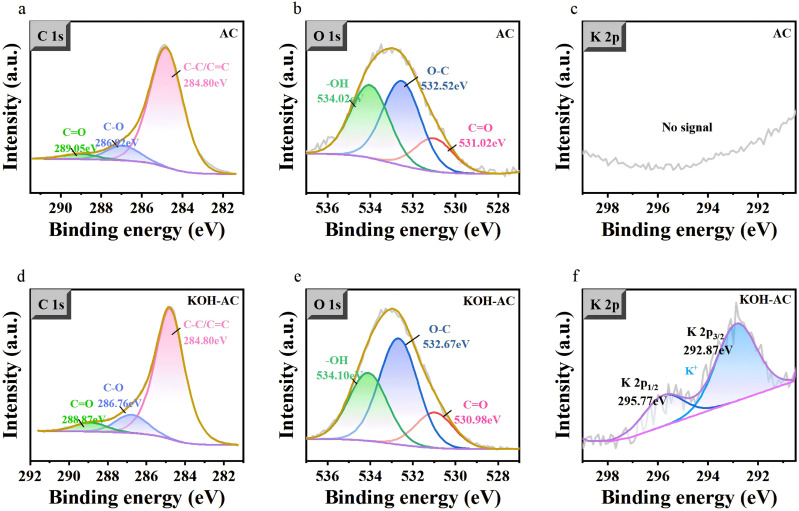
High-resolution XPS spectra comparison of AC and KOH-AC: (a, d) C 1s, (b, e) O 1s, (c, f) K 2p.

X-ray photoelectron spectroscopy (XPS) analysis further provided direct evidence for the surface chemical changes. As shown in Fig 5, compared with AC, the high-resolution C 1s spectrum of KOH-AC exhibited significantly increased intensities of the C-O and C = O peaks, indicating that the KOH activation introduced more abundant oxygen-containing functional groups [38]. The O 1s spectrum also showed a more complex oxygen-containing species. More importantly, the K 2p spectrum of KOH-AC showed a clear K^+^ characteristic peak, while there was none in AC, confirming the presence of potassium species. These increased oxygen-containing functional groups and the characteristic of residual K^+^ are consistent with the FTIR results, jointly enhancing the electrode’s hydrophilicity and providing additional ion exchange and pseudocapacitive active sites, thereby synergistically improving the chemical adsorption capacity of chloride ions.

#### 3.1.4 Electrochemical performance.

**Fig 6:**
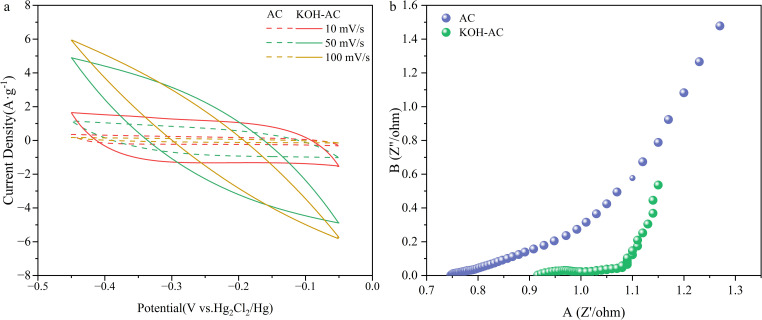
Electrochemistry results of the eletrode: (a) cyclic voltammetry; (b) electrochemical impedance spectroscopy.

To evaluate the electrochemical behavior of the AC and KOH-AC electrodes, cyclic voltammetry (CV) was employed. Fig 6a presents the CV curves at three representative scan rates of 10, 50, and 100 mV/s (the curves covering the full range of scan rates from 5 to 125 mV/s are shown in S3 Fig). The CV curve of the AC electrode showed lower rectangularity and a weaker current response, with more obvious polarization as the scanning rate increased. In contrast, the KOH-AC electrode exhibits a more ideal quasi-rectangular CV curve under all scanning rates, with stronger current response and faster potential reversal capability. This indicates its better double-layer capacitance characteristics, rate performance, and rapid ionic response [39]. At a scanning rate of 10 mV/s, the retention rates of rectangularity for the KOH-AC and AC electrodes were 97% and 88%, respectively; when the scanning rate increased to 100 mV/s, the retention rates were 88% and 70% respectively, indicating that the KOH-AC electrode has better ionic accessibility and structural stability. Even at a high scanning rate of 125 mV/s (S3 Fig), the capacitance retention rate of the KOH-AC electrode reaches 82%, significantly higher than that of the AC electrode at 63%. This is mainly attributed to the higher specific surface area and more developed pore size distribution of the KOH-AC electrode, providing better diffusion paths for electrolyte ions. These reversible surface redox reactions contribute to Cl^-^ removal through a pseudocapacitive mechanism, where charge transfer is coupled with ion adsorption/desorption, leading to enhanced adsorption capacity beyond the pure double-layer capacitance [11].

The electrochemical activity enhancement of KOH-AC was verified by electrochemical impedance spectroscopy (EIS). The EIS shows that the solution resistance (Rs) of KOH-AC (0.94 Ω) is comparable to that of pristine AC (0.79 Ω) (Table 2), indicating that electrolyte resistance remains essentially unchanged after activation [40]. However, the charge transfer resistance (Rct) is significantly reduced from approximately 0.2 Ω for AC to below 0.1 Ω for KOH-AC, as evidenced by the nearly negligible semicircle in the high-frequency region (Fig 6b), demonstrating markedly accelerated interfacial charge transfer kinetics. In the low-frequency region, the KOH-AC curve approaches a more vertical line, suggesting more ideal capacitive behavior and facilitated ion diffusion [35]. Consistently, the results reveal substantially enlarged integral areas and specific capacitances for KOH-AC across all scan rates. At 5 mV/s, the specific capacitance increased from 8.4 F/g (AC) to 18.7 F/g (KOH-AC), representing more than a twofold enhancement, and remains consistently higher even at 125 mV/s (10.6 F/g vs 4.3 F/g). The improved rate performance further indicates reduced diffusion limitation and enhanced ion accessibility [19]. These results demonstrate that KOH activation optimizes the pore structure and surface physicochemical properties of activated carbon, thereby promoting electron transport, lowering interfacial resistance, and significantly enhancing electrochemical activity.

**Table 2:** Capacitive performance of AC and KOH-AC under different scanning rates.

Scanning rate (mV/s)	AC		KOH-AC	
	Integral area (A·V)	Capacitor (F/g)	Integral area (A·V)	Capacitor (F/g)
5	0.0182	8.4	0.0257	18.7
10	0.0325	7.9	0.0468	17.6
25	0.0742	6.7	0.1081	15.2
50	0.1285	5.6	0.1915	13.3
75	0.1791	5.0	0.2653	12.2
100	0.2263	4.6	0.3382	11.3
125	0.2698	4.3	0.4015	10.6

### 3.2 Electrostatic adsorption performance

#### 3.2.1 Different voltage conditions.

**Fig 7:**
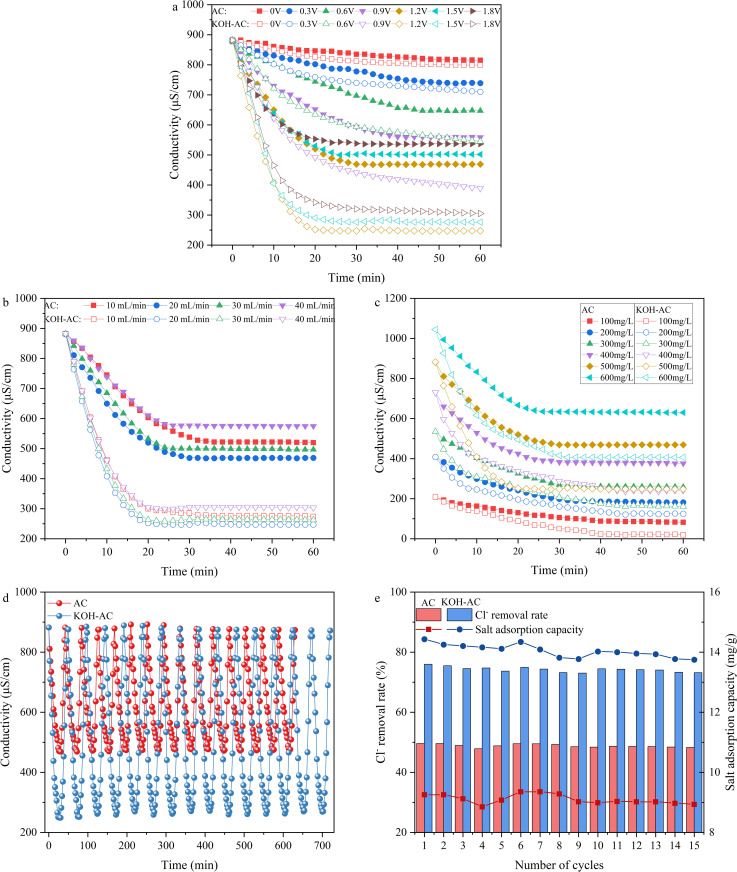
Electrical conductivity changes of modified activated carbon under different conditions: (a) voltage, (b) influent flow rate, (c) influent concentration; cyclic electrosorption-desorption performance: (d) electrical conductivity, (e) Cl^-^ removal and salt adsorption capacity.

As shown in Fig 7a, the electrosorption performance of KOH-AC and AC was significantly different under different applied voltages. In the absence of an applied potential, the ion removal process relies on physical adsorption. As the voltage increased, the conductivity rate of the KOH-AC electrode decreased substantially. The low voltage of 0.3 V limits ion migration, resulting in a conductivity attenuation of only 19.5%, which indicates low desalination efficiency at low voltages. The conductivity decay rate increases drastically to 72.0% when the voltage is raised to 1.2 V. The superior performance at 1.2 V arises from a balanced interplay between sufficient electrosorption driving force and minimized Faradaic side reactions, enabling efficient pore utilization and stable charge storage. The efficient utilization of electric fields can stimulate the directed migration of cations and anions, thereby promoting the formation of electric double layers [12]. However, when the voltage was increased to 1.8 V, the rate of conductivity decay decreased, which was attributed to water decomposition and other side reactions that increased energy consumption and reduced system stability. Therefore, KOH-AC exhibited optimal desalination performance at 1.2 V.

#### 3.2.2 Different flow rate conditions.

Flow rate primarily affected the thickness of the ion mass transfer boundary layer and the effective contact area at the electrode reaction interface [41]. Fig 7b showed the changes in conductivity under different influent flow rates. Compared with AC, KOH-AC exhibited superior electrosorption performance across the flow rate range of 10 ~ 40 mL/min, with the conductivity decay rate reaching a maximum of 72.0% at 20 mL/min. However, the conductivity decay rate was lowest when the flow rate was increased to 40 mL/min. Excessively high flow rates shortened the residence time of ions between electrodes, resulting in incomplete adsorption. BET analysis demonstrated that KOH-AC was much more effective at enlarging the micropores and mesopores, thereby reducing the ion transport route and internal diffusion resistance.

Moreover, the incorporation of oxygen-containing functional groups improves wettability between the electrode and electrolyte, optimizing charge distribution between electrodes to accelerate ion response and cycling capability [42].

#### 3.2.3 Different influent concentration conditions.

Fig 7c displayed the variation in conductivity of AC and KOH-AC during the desalination process at different initial concentrations. The results showed that KOH-AC had stronger conductivity decay than AC through the 100 ~ 600 mg/L. As time increased, the conductivity rapidly decreased and then leveled off. The conductivity decay of KOH-AC at 100 and 200 mg/L was much more than the decay of AC. Furthermore, KOH-AC maintained high desalination efficiency at 500 and 600 mg/L, while exhibiting a significantly reduced decrease in AC conductivity. This indicates that the modified electrode retained excellent adsorption performance under high salt loading conditions. Moreover, the conductivity decay rate accelerated with rising initial concentration, demonstrating that higher ion concentrations speed up double-layer formation and improve desalination efficiency. In summary, KOH-AC optimised the microporous and mesoporous structure of the electrode and introduced oxygen-containing functional groups to strengthen the interaction between the electrode and ions, which enhanced the electroadsorption process.

#### 3.2.4 Electrosorption-desorption cycling performance.

To evaluate the long-term cycling stability of the KOH-AC electrode, 15 consecutive adsorption – desorption cycle tests were conducted (Fig 7d). During the cycling process, there were significant differences in the conductivity changes between the KOH-AC and the original activated carbon (AC). Under the same operating conditions, the conductivity curve of KOH-AC remained consistently lower than that of the original activated carbon throughout all the cycle periods. The efficient chloride ion removal ability of KOH-AC led to a rapid decrease in conductivity, thereby enhancing the desalination efficiency. In contrast, the conductivity of the original activated carbon decreased at a slower rate, and the lowest conductivity value was significantly higher, reflecting its limited active sites and insufficient ion storage capacity.

Fig 7e further confirmed that after 15 cycles, the chloride ion removal of the AC decreased from 49.5% to 47.8%, the salt adsorption capacity (SAC) decreased from 9.25 mg/g to 8.73 mg/g (94.38% of the initial capacity), and the chloride ion adsorption capacity decreased from 5.62 mg/g to 5.30 mg/g. In KOH-AC, the chloride ion removal decreased from 75.9% to 72.9%, the SAC decreased only from 14.43 mg/g to 13.70 mg/g (94.69% of the initial capacity), and the chloride ion adsorption capacity decreased from 5.876 mg/g to 8.32 mg/g. Although the capacity retention of both electrodes remained above 94%, the absolute SAC and chloride ion removal efficiency of KOH-AC were consistently higher than those of the AC. Compared with other studies, the adsorption performance was markedly superior to that previously reported (S3 Table). This quantitative comparison indicates that the KOH modification has increased the electrode’s adsorption capacity by more than 50% while maintaining the excellent cycling stability of the material. This further demonstrates the superiority of the KOH activation strategy in improving the capacitance dechlorination performance.

### 3.3 Regression models for Cl^-^ removal prediction

#### 3.3.1 Machine learning prediction model.

**Fig 8:**
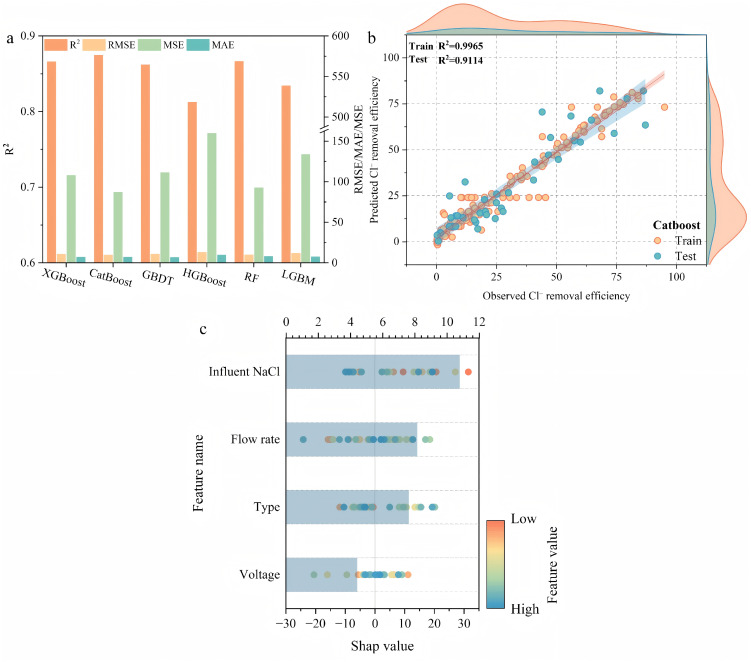
(a) Prediction parameters of machine learning models for chloride ion removal; (b) Actual versus predicted chloride ion removal data obtained from CatBoost; (c) SHAP value analysis of the CatBoost model for each feature variable affecting chloride ion removal.

Machine learning models were employed to predict chloride ion removal. As shown in Fig 8a, the six models (XGBoost, CatBoost, GBDT, HGBoost, RF, and LGBM) exhibited a significant disparity in the main metric of performance, such as R^2^, RMSE, MSE and MAE. Among them, the CatBoost model was the best performer overall, as it had the highest R^2^ value and the lowest RMSE, MSE and MAE values. This implies that there is a better fit, fewer errors in prediction and greater stability. Fig 8b also demonstrates the quality of CatBoost model in terms of both fitting and predicting, the training and test set R^2^ of the model are 0.9965 and 0.9114 respectively. This reflects a strong generalization skill and successful evading overfitting. Predicted values exhibit high linear correlation with observed values, with data points uniformly distributed near the fitted line, confirming the model accurately captures trends in chloride ion removal efficiency. Overall, the CatBoost model outperforms other ensemble learning algorithms in terms of accuracy, robustness, and generalization capability.

#### 3.3.2 Feature importance.

The interpretability of the machine learning model was evaluated using the Shapley Additivity for Prediction (SHAP) method, which provides a quantitative measure of the relative contribution of each input feature to the prediction of chloride ion removal efficiency. As illustrated in Fig 8c, the features were ranked as follows: influent Nacl concentration > flow rate > electrode material type > voltage. The average shap value of the influent NaCl concentration was the highest and most influential variable in the model predictions. This highlights the significant impact of changes in ion strength and conductivity on the electrosorption process [23]. The flow rate was second, meaning that hydrodynamic conditions significantly influenced proton transfer and ion diffusion at the electrode surface [43]. Stable flow velocities reduce concentration polarisation and promote ion migration. An unsuitable flow rate can inhibit adsorption to an adequate degree. The influence of electrode material type is relatively insignificant. Meanwhile, the total effect of voltage was comparatively negligible, but its role in controlling the strength of the electric field and the electrochemical driving forces cannot be overlooked. In general, Shap analysis proved to be an effective method of explaining the comparative impact of operating parameters and material properties on Catboost prediction results. This demonstrates that the Catboost model is a rational response to the combined effect of multiple factors during chloride ion extraction.

## 4. Conclusion

This study systematically investigates the performance and mechanism of KOH-activated activated carbon electrodes in high-chlorine wastewater treatment. The results reveal that KOH activation optimizes the microstructure and surface chemistry of activated carbon, increasing its specific surface area from 104.48 m^2^/g to 1390.88 m^2^/g with a micropore surface area of 985.51 m^2^/g, and forming a hierarchical pore structure with 4.3 nm mesopores as the main transport channels. Meanwhile, the abundant oxygen-containing functional groups and structural defects introduced during activation endow the material with excellent electrochemical properties. The KOH-AC electrode exhibits outstanding ionic diffusion and electrochemical transport characteristics, with a capacitance retention rate of 81.5% and a charge transfer resistance below 0.1 Ω. Under the optimal conditions (1.2 V, 20 mL/min), it achieves a chloride adsorption capacity of approximately 14.43 mg/g and a chloride removal efficiency of 76%, while maintaining remarkable stability in adsorption-desorption cycles. Furthermore, the established CatBoost machine learning model enables efficient prediction of chloride ion removal with an R^2^ value of 0.9114, and SHAP analysis identifies the priority of influencing factors as follows: influent NaCl concentration, flow rate, electrode material type, and applied voltage. Overall, the high-performance CDI electrodes developed here provide new insights for dechlorination of coal terminal wastewater. However, current research remains at the laboratory scale, and further studies are required to evaluate energy consumption, electrode lifespan, fouling resistance, and system integration for engineering applications.

## Supporting information

S1 Fig Variation of solution conductivity of electrodes at different PTFE solid content. (TIF)

S2 Fig Fitting curves of Cl^-^ concentration and conductivity. (TIF)

S3 Fig Cyclic voltammograms at different rates. (TIF)

S1 Table CDI parameters. (PDF)

S2 Table Corresponding conductivity values of Cl^-^ concentration of different concentrations. (PDF)

S3 Table Compared with other studies on salt removal. (PDF)

S1 Text (PDF)

## References

[1] Aubourg MA, Livi KJT, Sawtell GG, Sanchez-Gonzalez CC, Spada NJ, Dickerson RR, et al. Use of electron microscopy to determine presence of coal dust in a neighborhood bordering an open-air coal terminal in Curtis Bay, Baltimore, Maryland, USA. Sci Total Environ. 2024;957:176842. doi: 10.1016/j.scitotenv.2024.176842 39396779 PMC11698221

[2] Chen L, Xiang H, Zhou LT, Zhang YQ, Ding YC, Wu D, et al. Low-voltage stimulated denitrification performance of high-salinity wastewater using halotolerant microorganisms. Bioresour Technol. 2024;401:130688. doi: 10.1016/j.biortech.2024.130688 38604298

[3] Feng H, Liao X, Yang R, Chen S, Zhang Z, Tong J, et al. Generation, toxicity, and reduction of chlorinated byproducts: overcome bottlenecks of electrochemical advanced oxidation technology to treat high chloride wastewater. Water Res. 2023;230:119531. doi: 10.1016/j.watres.2022.119531 36580803

[4] Arous F, Hamdi C, Bessadok S, Boudagga S, Aydi A, Li W, et al. Demonstration scale chemical–physical treatment and agricultural reuse of highly saline textile wastewater. Water Environ J. 2024;38(4):573–86. doi: 10.1111/wej.12946

[5] Uğan M, Onac C, Kaya A, Köseoğlu D, Akdoğan A. Removal of Reactive Red 195 dye from textile industry wastewater with Deep Eutectic Solvent-based green extraction. J Mol Liq. 2024;398:124249. doi: 10.1016/j.molliq.2024.124249

[6] Yang L-J, Guan H-Y, Yuan S, Sun T, Jiang A-N, Feng J-J. Research progress of chlorine corrosion resistance in seawater electrolysis: materials and technologies. Chem Eng J. 2025;503:158458. doi: 10.1016/j.cej.2024.158458

[7] Li H, Chen Y, Long J, Jiang D, Liu J, Li S, et al. Simultaneous removal of thallium and chloride from a highly saline industrial wastewater using modified anion exchange resins. J Hazard Mater. 2017;333:179–85. doi: 10.1016/j.jhazmat.2017.03.020 28355586

[8] Habib S, Weinman ST. A review on the synthesis of fully aromatic polyamide reverse osmosis membranes. Desalination. 2021;502:114939. doi: 10.1016/j.desal.2021.114939

[9] Duan L, Yun Q, Jiang G, Teng D, Zhou G, Cao Y. A review of chloride ions removal from high chloride industrial wastewater: sources, hazards, and mechanisms. J Environ Manage. 2024;353:120184. doi: 10.1016/j.jenvman.2024.120184 38310791

[10] Li H, Liu B, Pang F, Xu X, Xiao Z, Shang N, et al. Ultrafine Ag/AgCl heterostructures confined in the MXene nanosheets for enhanced chloride ion storage. Nano Energy. 2025;141:111135. doi: 10.1016/j.nanoen.2025.111135

[11] Ansari MZ, Ansari SA, Parveen N, Alam MW, Kim S-H. The role of high-entropy materials and d-band center adjustments in supercapacitor development. J Energy Storage. 2025;131:117535. doi: 10.1016/j.est.2025.117535

[12] Datar SD, Mohanapriya K, Ahirrao DJ, Jha N. Comparative study of electrosorption performance of solar reduced graphene oxide in flow-between and flow-through capacitive deionization architectures. Sep Purif Technol. 2021;257:117972. doi: 10.1016/j.seppur.2020.117972

[13] Hui B, Zhou H, Liu A, Fei C, Xu T, Chen C, et al. Nitrogen-rich self-doping modified porous carbon material as a CDI electrode for brine desalination. Carbon Lett. 2025;35(4):1701–14. doi: 10.1007/s42823-025-00891-w

[14] Zhang Y, Yue P, Zhang C, Wang Y, Wu X. Facile synthesis of three-dimensional interconnected porous carbon for high performance supercapacitor. Diam Relat Mater. 2023;136:109941. doi: 10.1016/j.diamond.2023.109941

[15] Chen R, Deng X, Wang C, Du J, Zhao Z, Shi W, et al. Enhanced desalination performances by using porous polyaniline-activated carbon composite flow-electrodes in capacitive deionization system. Desalination. 2023;557:116568. doi: 10.1016/j.desal.2023.116568

[16] Zhao C, Ge L, Mai L, Li X, Chen S, Li Q, et al. Review on coal-based activated carbon: preparation, modification, application, regeneration, and perspectives. Energy Fuels. 2023;37:11622–42. doi: 10.1021/acs.energyfuels.3c01866

[17] Alazmi A. Synergistic effect of hydrothermal and physical activation approaches to fabricate activated carbon for energy storage applications. Ceram Int. 2022;48:22131–40. doi: 10.1016/j.ceramint.2022.04.205

[18] Ansari SA, Parveen N, Ansari MZ, Alsulaim GM, Alam MW, Khan MY, et al. Exploring recent advances in the versatility and efficiency of carbon materials for next generation supercapacitor applications: a comprehensive review. Prog Mater Sci. 2025;154:101493. doi: 10.1016/j.pmatsci.2025.101493

[19] Williams NE, Oba OA, Aydinlik NP. Modification, production, and methods of KOH‐activated carbon. ChemBioEng Rev. 2022;9(1):164–89. doi: 10.1002/cben.202100030

[20] Han W, Wang H, Xia K, Chen S, Yan P, Deng T, et al. Superior nitrogen-doped activated carbon materials for water cleaning and energy storing prepared from renewable leather wastes. Environ Int. 2020;142:105846. doi: 10.1016/j.envint.2020.105846 32585500

[21] Domínguez JR, Durán-Valle CJ, Mateos-García G. Synthesis and characterisation of acid/basic modified adsorbents. Application for chlorophenols removal. Environ Res. 2022;207:112187. doi: 10.1016/j.envres.2021.112187 34634312

[22] Ma J, Liu Y, Chen S, Du Y, Wu H. Changes in the pore structure of modified sludge-activated carbon and its effect on the adsorption characteristics of CO2 under high pressure. Microporous Mesoporous Mater. 2022;345:112255. doi: 10.1016/j.micromeso.2022.112255

[23] Liu L, Wu J, Wang Z, Liu J, Sun X, Wang H. A novel cell structure for high performance electrosorption process. Desalination. 2025;601:118603. doi: 10.1016/j.desal.2025.118603

[24] Lu D, Ma X, Lu J, Qian Y, Geng Y, Wang J, et al. Ensemble machine learning reveals key structural and operational features governing ion selectivity of polyamide nanofiltration membranes. Desalination. 2023;564:116748. doi: 10.1016/j.desal.2023.116748

[25] Sukarno, Chong JY, Cong G. Predicting the boron removal of reverse osmosis membranes using machine learning. Desalination. 2024;586:117854. doi: 10.1016/j.desal.2024.117854

[26] Cheng X, Hu Z, Wang X, Li J, Wei B, Liu J, et al. Flue gas enhanced water leaching: Prediction and condition optimization of demineralization effect from coal via machine learning algorithm. Process Saf Environ Prot. 2023;178:247–54. doi: 10.1016/j.psep.2023.08.030

[27] Kong H, Gao M, Li R, Miao L, Kang Y, Xiao W, et al. Machine learning-based prediction of desalination capacity of electrochemical performance of nitrogen-doped for capacitive deionization. Desalination. 2025;607:118820. doi: 10.1016/j.desal.2025.118820

[28] Saffarimiandoab F, Mattesini R, Fu W, Kuruoglu EE, Zhang X. Insights on features’ contribution to desalination dynamics and capacity of capacitive deionization through machine learning study. Desalination. 2021;515:115197. doi: 10.1016/j.desal.2021.115197

[29] Soker O, Tajdini B, Abarca-Perez A, Wadia A, Bellona C, Hao S, et al. Reuse of spent granular activated carbon for PFAS removal following hydrothermal alkaline treatment. Water Res. 2025;283:123794. doi: 10.1016/j.watres.2025.123794 40378469

[30] McKague M, Fathiannasab H, Agnaou M, Sadeghi MA, Gostick J. Extending pore network models to include electrical double layer effects in micropores for studying capacitive deionization. Desalination. 2022;535:115784. doi: 10.1016/j.desal.2022.115784

[31] Quan C, Wang W, Su J, Gao N, Wu C, Xu G. Characteristics of activated carbon derived from Camellia oleifera cake for nickel ions adsorption. Biomass Bioenergy. 2023;171:106748. doi: 10.1016/j.biombioe.2023.106748

[32] Li G, Han J, Wang Y, Wu S, Ge L, An X, et al. Study of enhanced nitrogen removal performance and mechanism of iron-modified activated carbon in low-temperature environments. J Environ Chem Eng. 2025;13(3):116951. doi: 10.1016/j.jece.2025.116951

[33] Fu H, Zhang J, Zhao L, Huang Y, Chen B. Investigations of NO reduction by coal-based activated carbon with KOH activation: performance and mechanism. Chemosphere. 2024;346:140506. doi: 10.1016/j.chemosphere.2023.140506 37914046

[34] Han J, Hu X, Sun L, Wang Q, Ulbricht M, Lv L, et al. A novel γ-Fe3O4-N-BC combined membrane bioreactor for wastewater treatment: performance and mechanism. Sep Purif Technol. 2024;336:126330. doi: 10.1016/j.seppur.2024.126330

[35] Zhai Z, Wang S, Xu Y, Zhang L, Wang X, Yu H, et al. Starch-based carbon aerogels prepared by an innovative KOH activation method for supercapacitors. Int J Biol Macromol. 2024;257(Pt 1):128587. doi: 10.1016/j.ijbiomac.2023.128587 38065463

[36] Leng L, Xu S, Liu R, Yu T, Zhuo X, Leng S, et al. Nitrogen containing functional groups of biochar: an overview. Bioresour Technol. 2020;298:122286. doi: 10.1016/j.biortech.2019.122286 31690478

[37] Ojo BO, Arotiba OA, Mabuba N. Coupling piezo-polarization effect on Ti/BaZrTiO3 anode with sonoelectro-Fenton oxidation for the removal of aspirin in wastewater. Electrochim Acta. 2023;459:142501. doi: 10.1016/j.electacta.2023.142501

[38] Parveen N. Enhanced energy storage using bio-waste derived carbon and three-dimensional NiCo2O4 structures in asymmetric supercapacitors. J Ind Eng Chem. 2025;150:824–36. doi: 10.1016/j.jiec.2025.06.027

[39] Ansari SA, Alam MW, BaQais A, Yewale MA. Exploring the effective electrochemical diclofenac sensing, energy storage, and photocatalytic capabilities of strontium-doped molybdenum oxide nanoparticles. J Ind Eng Chem. 2026;155:788–800. doi: 10.1016/j.jiec.2025.08.033

[40] Ansari SA. Fabrication and evaluation of binder-free metal-molybdate electrodes for improved energy storage and hydrogen evolution applications. J Power Sources. 2025;646:237185. doi: 10.1016/j.jpowsour.2025.237185

[41] Feng S, Huang K. Interfacial salt effect induced by competitive adsorption of coexisting ions: a new understanding of the mass transfer selectivity in the near-interface boundary layer. Sep Purif Technol. 2024;336:126244. doi: 10.1016/j.seppur.2023.126244

[42] Han J, Wang Q, Sun L, Ulbricht M, Fischer L, Yang H, et al. Moderate oxidation–coagulation pretreatment for mitigating ultrafiltration membrane fouling by algae-laden water: performance and mechanisms. Desalination. 2026;619:119522. doi: 10.1016/j.desal.2025.119522

[43] Liu C, Ma X, Ma L, Xu Y, Wang F, Huang L, et al. A novel asymmetric CDI device for targeted removal of cation in water desalination. Environ Technol. 2023;44(11):1626–41. doi: 10.1080/09593330.2021.2010129 34807812

