# Supplementary figures and images for: KOH activation of carbon electrodes for enhanced capacitive dechlorination: Performance and mechanisms
Source: PLoS One. 2026 May 27;21(5):e0347780. doi: 10.1371/journal.pone.0347780 (PMC13215479; doi:10.1371/journal.pone.0347780)

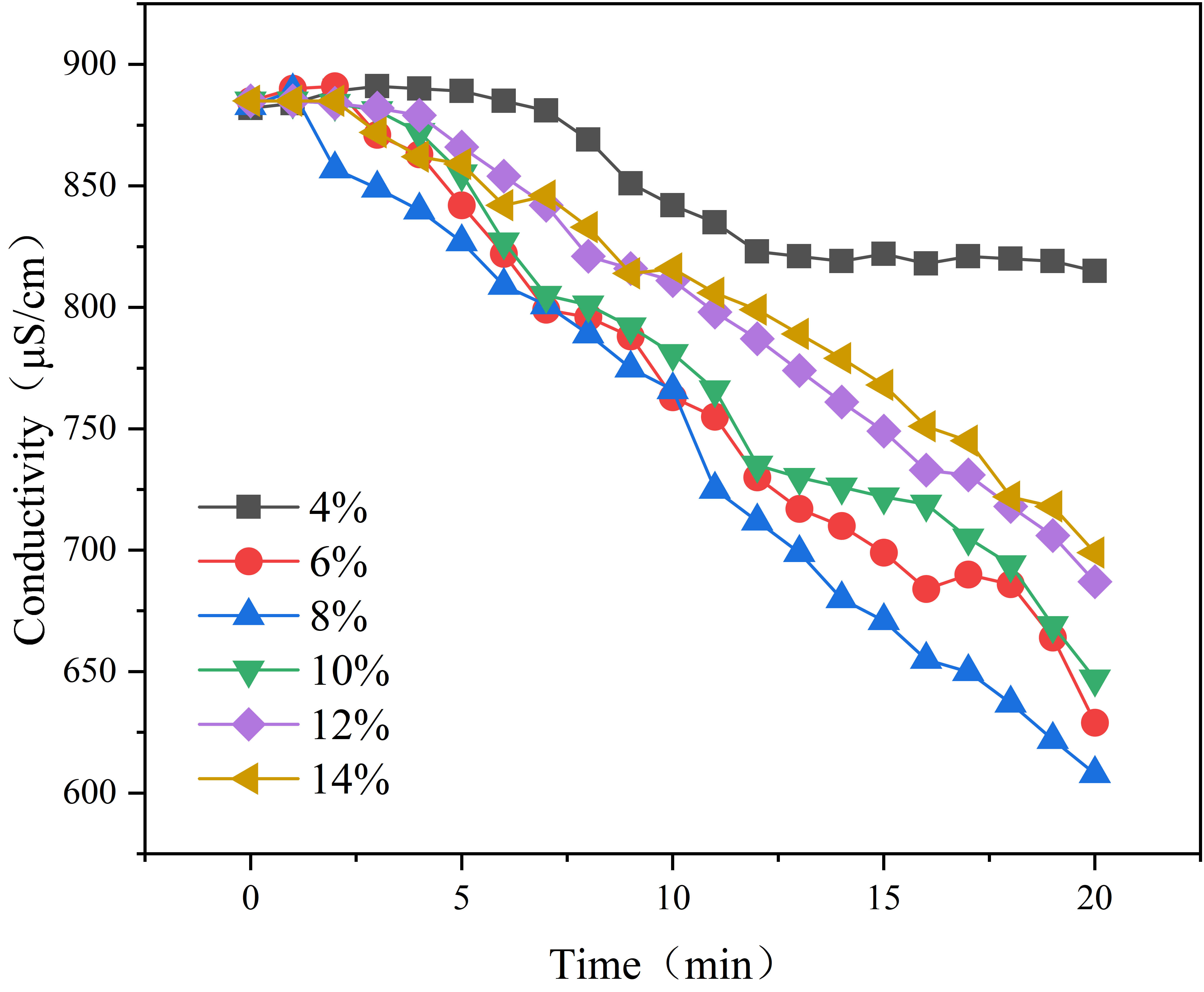

Supplement: S1 Fig — (TIF) [file pone.0347780.s001.tif]

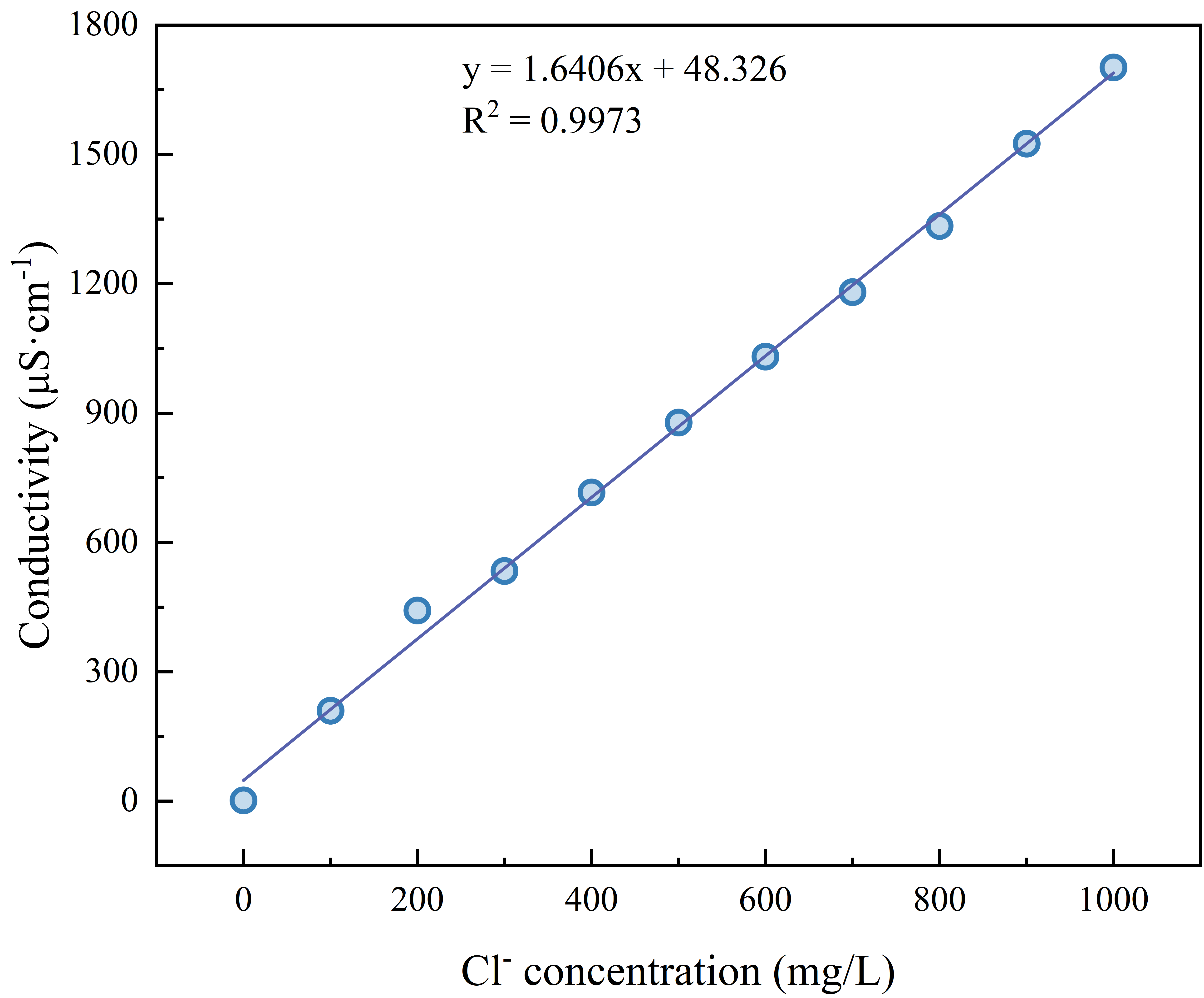

Supplement: S2 Fig — (TIF) [file pone.0347780.s002.tif]

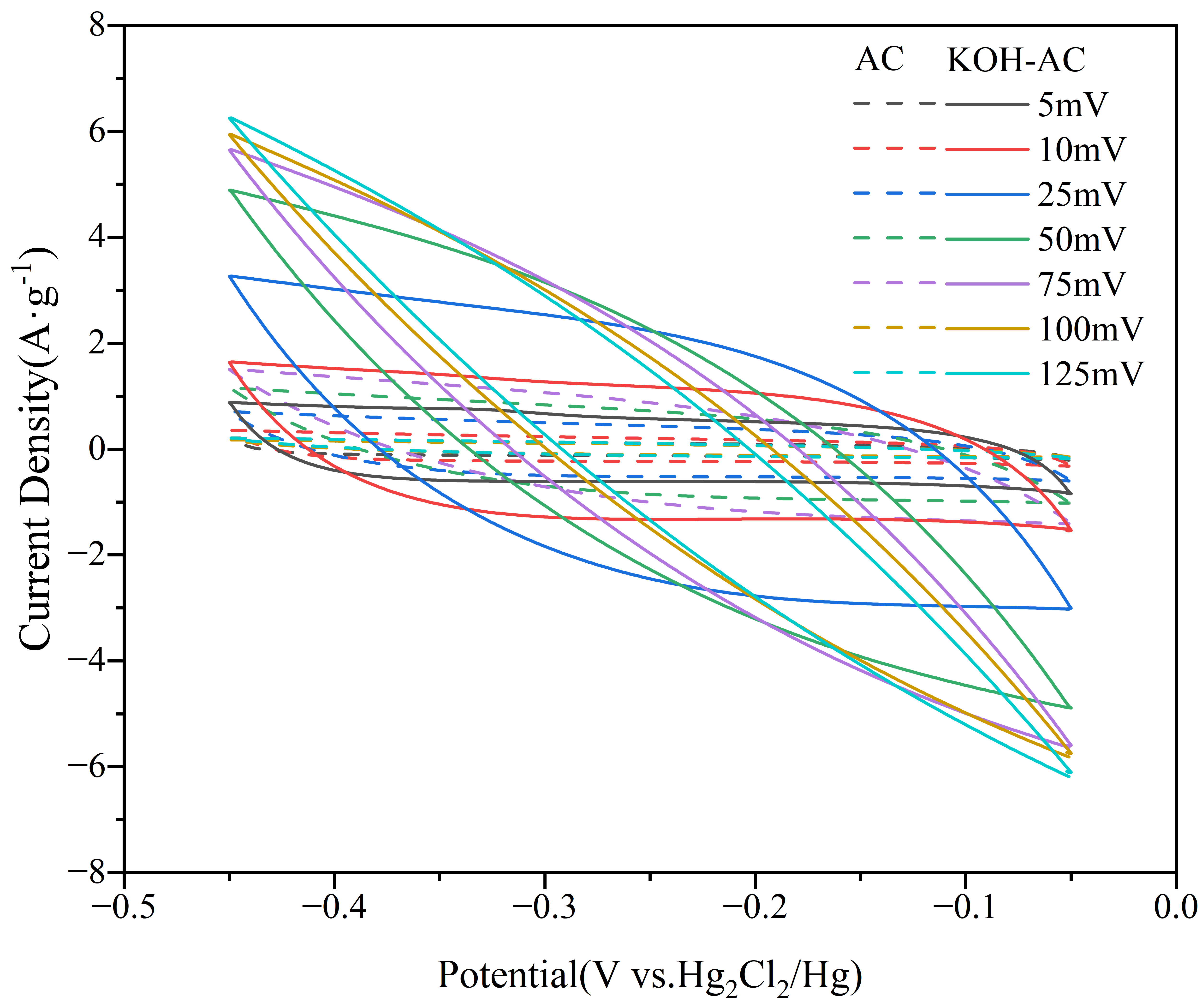

Supplement: S3 Fig — (TIF) [file pone.0347780.s003.tif]
