# Supplementary material for: KOH activation of carbon electrodes for enhanced capacitive dechlorination: Performance and mechanisms
Source: PLoS One. 2026 May 27;21(5):e0347780. doi: 10.1371/journal.pone.0347780 (PMC13215479; doi:10.1371/journal.pone.0347780)
Supplement: S1 Table — (PDF) [file pone.0347780.s004.pdf]

Table S1 CDI Parameters

CDI	Parameter
Type	CD5050
Size	25 cm^3
Length and width	9*9 cm
Height	3.5 cm
Internal volume	7.5 ml
Distance between positive and negative electrodes	2 mm
Liquid pipe joint	M5-3.8
Volume of electrolyte solution	150 ml
