# Supplementary material for: KOH activation of carbon electrodes for enhanced capacitive dechlorination: Performance and mechanisms
Source: PLoS One. 2026 May 27;21(5):e0347780. doi: 10.1371/journal.pone.0347780 (PMC13215479; doi:10.1371/journal.pone.0347780)
Supplement: S2 Table — (PDF) [file pone.0347780.s005.pdf]

Table S2 Corresponding conductivity values of Cl^- concentration of different concentrations

Cl^- (mg/L)	0	100	200	300	400	500	600	800	900	1000
Conductivity (µs/cm)	1.74	210	442	534	716	878	1031	1334	1525	1702
