# Supplementary material for: KOH activation of carbon electrodes for enhanced capacitive dechlorination: Performance and mechanisms
Source: PLoS One. 2026 May 27;21(5):e0347780. doi: 10.1371/journal.pone.0347780 (PMC13215479; doi:10.1371/journal.pone.0347780)
Supplement: S3 Table — (PDF) [file pone.0347780.s006.pdf]

Table S3 Compared with other studies on salt removal

Electrode material	Modification method	Salt removal rate	Reference
Nanoporous activated carbon cloth (ANCC)	$KOH+HNO_3$ etching	67% (DR)	[1]
Activated carbon powder	Nitric acid modification	~69% (DR, from ~54%)	[2]
Activated carbon fiber (ACF)	Nitric acid treatment	>74% (Charge efficiency)	[3]
Powdered activated carbon	KOH modification	54.64% (DR at 1.2V)	[4]
Porous carbon from jackfruit peels	KOH activation (700 °C)	20% (DR at 500 mg/L, 2.0 V)	[5]
Walnut shell biochar (WSC)	KOH activation	47.82% (DR)	[6]
OH/TiO_2-AC composite	KOH + Sol-gel (TiO_2)	71.15% (DR)	[7]
Activated carbon from natural waste	$H_2SO_4+NaNO_3+KMnO_4$ activation	55% (DR)	[8]
Spent activated carbon (AC-spent)	$KOH/H_3PO_4/ZnCl_2/NaOH$ regeneration	57.3% (DR)	[9]
	KOH-AC	75.9% (DR)	This study

[1] H. Oh, J. Lee, H. Ahn, Y. Jeong, Y. Kim, C. Chi, Nanoporous activated carbon cloth for capacitive deionization of aqueous solution, Thin Solid Films, 515 (2006) 220-225.

https://doi.org/10.1016/j.tsf.2005.12.146.

[2] W. Huang, Y. Zhang, S. Bao, R. Cruz, S. Song, Desalination by capacitive deionization process using nitric acid-modified activated carbon as the electrodes, Desalination, 340 (2014) 67-72. https://doi.org/10.1016/j.desal.2014.02.012.

[3] T. Wu, G. Wang, Q. Dong, B. Qian, Y. Meng, J. Qiu, Asymmetric capacitive deionization utilizing nitric acid treated activated carbon fiber as the cathode, Electrochim Acta, 176 (2015) 426-433. https://doi.org/10.1016/j.electacta.2015.07.037.

[4] S. Jiang, D. Ma, G. Sheng, S. Jiang, M. Chen, Research on the capacitive deionization performance of activated carbon-coated electrodes modified with KOH, Ind. Water Treat., 35 (9) (2015) 53-56. https://kns.cnki.net/kcms/detail/detail.aspx?dbcode=CJFD&filename=GYSB201509015

[5] J. Elisadiki, Y.A.C. Jande, R.L. Machunda, T.E. Kibona, Porous carbon derived from Artocarpus heterophyllus peels for capacitive deionization electrodes, Carbon, 147 (2019) 582-593. https://doi.org/10.1016/j.carbon.2019.03.036.

[6] Y. Wei, R. Shi, H. Zhao, K. Li, Z. Guo, Y. Chang, M. Shen, KOH Activated Walnut Shell Biochar Electrode of Capacitive Deionization and Its Desalination Performance, J. Electrochem. Soc., 171 (2024) 113501. https://doi.org/10.1149/1945-7111/ad8968.

[7] Q. Weng, H. Chen, S. He, Y. Zhang, Y. Yang, Research on the performance of plate and roll capacitor deionization devices based on supercapacitor carbon electrodes, Zhejiang Univ. Sci, 51 (5) (2024) 521-530. https://doi.org/10.3785/j.issn.1008-9497.2024.05.001

[8] G.A. Elawadi, Low-Energy Desalination Techniques, Development of Capacitive Deionization Systems, and Utilization of Activated Carbon, Materials, 17 (2024) 5130.

https://doi.org/10.3390/ma17205130.

[9] A.S. Idrissi, Y. Seffar, A.E. Bendali, J. Alami, H. Sehaqui, M. Dahbi, Recovery of spent activated carbon from treatment plants for high performance electrodes in capacitive deionization processes, Electrochim Acta, 513 (2025) 145446. https://doi.org/10.1016/j.electacta.2024.145446.
