# Supplementary material for: KOH activation of carbon electrodes for enhanced capacitive dechlorination: Performance and mechanisms
Source: PLoS One. 2026 May 27;21(5):e0347780. doi: 10.1371/journal.pone.0347780 (PMC13215479; doi:10.1371/journal.pone.0347780)
Supplement: S1 Text — (PDF) [file pone.0347780.s007.pdf]

Text S1

All chemical reagents and materials used in this experiment were either of analytical grade or met specified purity standards, with their detailed sources as follows: activated carbon (AR) and hydrochloric acid (HCl, AR) were both purchased from Sinopharm Chemical Reagent Co., Ltd.; conductive carbon black, polytetrafluoroethylene (PTFE, 60% solid content), and nickel foam sheets were supplied by Suzhou Shengnuo Technology Co., Ltd.; N-methylpyrrolidone (NMP, AR) was obtained from Fuchen (Tianjin) Chemical Reagent Co., Ltd.; anhydrous ethanol (AR) and sodium chloride (NaCl, AR) were acquired from Tianjin Fengchuan Chemical Reagent Technology Co., Ltd.; and potassium hydroxide (KOH, AR) was provided by Tianjin Hengxing Chemical Reagent Manufacturing Co., Ltd.
